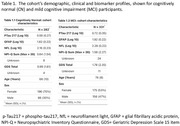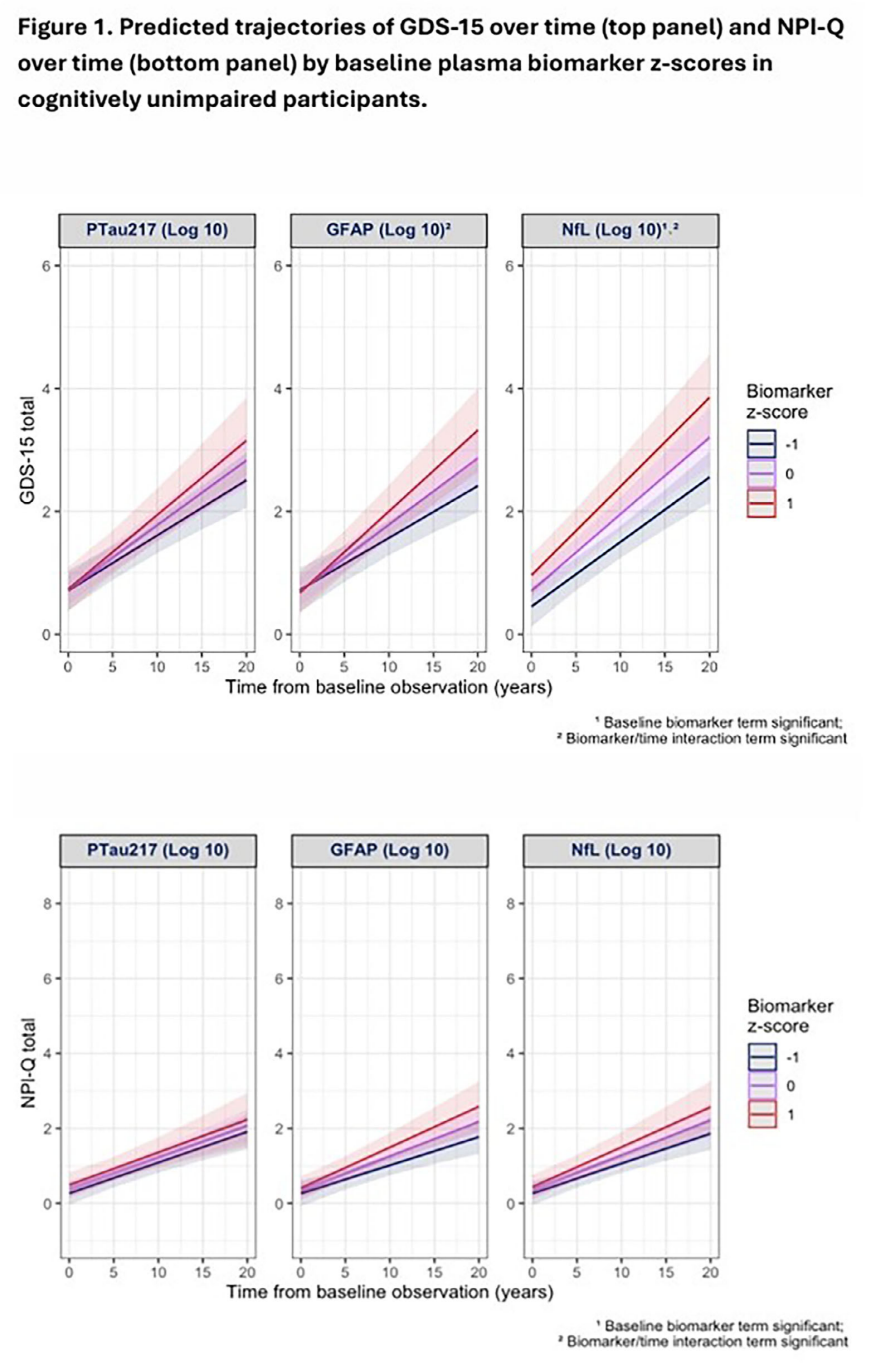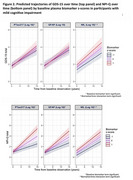# Associations between plasma biomarkers and longitudinal neuropsychiatric symptoms across the cognitive spectrum in older adults: findings from the Massachusetts Alzheimer's Disease Research Center

**DOI:** 10.1002/alz70857_103645

**Published:** 2025-12-25

**Authors:** Jennifer R. Gatchel, Kexin Yu, Phebe Palmer, Jacqueline Beltran, Chao‐Yi Wu, Hyun‐Sik Yang, Pia Kivisäkk, Deborah Blacker, Steven E Arnold, Gad A. Marshall, Hiroko H Dodge

**Affiliations:** ^1^ Massachusetts General Hospital, Harvard Medical School, Boston, MA, USA; ^2^ Athinoula A. Martinos Center for Biomedical Imaging, Charlestown, MA, USA; ^3^ McLean Hospital, Belmont, MA, USA; ^4^ University of Texas Southwestern Medical Center, Dallas, TX, USA; ^5^ Massachusetts General Hospital, Boston, MA, USA; ^6^ Harvard Medical School, Boston, MA, USA; ^7^ Department of Neurology, Massachusetts General Hospital/Brigham & Women's Hospital, Boston, MA, USA; ^8^ Center for Alzheimer Research and Treatment, Department of Neurology, Brigham and Women's Hospital, Boston, MA, USA; ^9^ Department of Neurology, Harvard Medical School, Boston, MA, USA; ^10^ Brigham and Women's Hospital, Boston, MA, USA; ^11^ Massachusetts Alzheimer's Disease Research Center, Charlestown, MA, USA; ^12^ Department of Epidemiology, Harvard T. H. Chan School of Public Health, Boston, MA, USA; ^13^ MassGeneral Institute for Neurodegenerative Disease, Charlestown, MA, USA

## Abstract

**Background:**

Neuropsychiatric symptoms (NPS) are common in the preclinical and prodromal stages of Alzheimer's Disease and Related Dementias (ADRD) and are associated with cognitive and functional decline. However, the neurobiology of these symptoms and prognostic markers of their emergence are not well understood. We sought to examine the associations between established baseline plasma biomarkers of ADRD and the longitudinal trajectory of NPS in older adults.

**Method:**

Participants were from the Massachusetts Alzheimer's Disease Research Center longitudinal research cohort. A subset, spanning consensus diagnoses of cognitively normal (CN)(*n* = 282, age:68±10) years) to mild cognitive impairment (MCI)(*n* = 331, age:74±8 years), had baseline plasma samples assayed for phospho‐tau^217^ (*p*‐tau^217^), neurofilament‐light (NfL) and glial fibrillary acidic protein (GFAP) (Table 1). Average follow‐up was 8.6±4.2 years (CN) and 7.2±4.1 years (MCI). NPS were measured annually using the self‐reported Geriatric Depression Scale (GDS‐15) and the study‐partner‐reported Neuropsychiatric Inventory Questionnaire (NPI‐Q). Plasma biomarkers were log‐transformed and z‐scores were derived using baseline values of the study sample. In longitudinal linear mixed‐effects models, we examined the NPS measures as the dependent variable in relation to individual plasma biomarkers, time, and the plasma biomarker × time interaction, controlling for age and sex.

**Result:**

In CN participants, plasma NfL and GFAP were associated with change in GDS (NfL x time: β=0.02, CI=0.01‐0.03 *p* = 0.007); GFAP x time: β=0.02, CI=0.01‐0.04, *p* = 0.002) but not with NPI‐Q (*p* >0.05 for all); (Figure 1). In MCI participants, *p*‐tau^217^ and NfL were associated with change in GDS (*p*‐tau^217^: β=0.02, CI=0‐0.04, *p* = 0.04; NfL: β=0.02, CI=0‐0.04, *p* = 0.02) whereas all three biomarkers predicted increasing NPI‐Q score over time (*p*‐tau^217^: β=0.09 CI=0.07‐0.12, *p* <0.001; NfL: β=0.03, CI=0‐0.06 *p* = 0.025; GFAP: β=0.07, CI=0.04‐0.10, *p* <0.001) (Figure 2). In exploratory NPI‐Q analyses, when NfL and GFAP were individually added as predictors alongside p*‐*tau^217^, only *p*‐tau^217^ remained significant.

**Conclusion:**

Findings suggest differential plasma biomarker‐NPS associations across the early ADRD spectrum, with a central role of *p*‐tau^217^ at the stage of MCI. Results also support biomarker signatures of affective vs. overall NPS. Further dissecting plasma biomarker signatures of specific NPS domains will provide insight into the prognostic value of plasma measures in predicting NPS trajectories over the course of ADRD.